# Completion of draft bacterial genomes by long-read sequencing of synthetic genomic pools

**DOI:** 10.1186/s12864-020-06910-6

**Published:** 2020-07-29

**Authors:** Hooman Derakhshani, Steve P. Bernier, Victoria A. Marko, Michael G. Surette

**Affiliations:** 1 Farncombe Family Digestive Health Research Institute, McMaster University, Hamilton, Ontario Canada; 2 Department of Medicine, McMaster University, Hamilton, Ontario Canada; 3 Department of Biochemistry and Biomedical Sciences, McMaster University, Hamilton, Ontario Canada

**Keywords:** De novo assembly, Bacterial genomics, Hybrid assembly, Synthetic genomic pool

## Abstract

**Background:**

Illumina technology currently dominates bacterial genomics due to its high read accuracy and low sequencing cost. However, the incompleteness of draft genomes generated by Illumina reads limits their application in comprehensive genomics analyses. Alternatively, hybrid assembly using both Illumina short reads and long reads generated by single molecule sequencing technologies can enable assembly of complete bacterial genomes, yet the high per-genome cost of long-read sequencing limits the widespread use of this approach in bacterial genomics. Here we developed a protocol for hybrid assembly of complete bacterial genomes using miniaturized multiplexed Illumina sequencing and non-barcoded PacBio sequencing of a synthetic genomic pool (SGP), thus significantly decreasing the overall per-genome cost of sequencing.

**Results:**

We evaluated the performance of SGP hybrid assembly on the genomes of 20 bacterial isolates with different genome sizes, a wide range of GC contents, and varying levels of phylogenetic relatedness. By improving the contiguity of Illumina assemblies, SGP hybrid assembly generated 17 complete and 3 nearly complete bacterial genomes. Increased contiguity of SGP hybrid assemblies resulted in considerable improvement in gene prediction and annotation. In addition, SGP hybrid assembly was able to resolve repeat elements and identify intragenomic heterogeneities, e.g. different copies of 16S rRNA genes, that would otherwise go undetected by short-read-only assembly. Comprehensive comparison of SGP hybrid assemblies with those generated using multiplexed PacBio long reads (long-read-only assembly) also revealed the relative advantage of SGP hybrid assembly in terms of assembly quality. In particular, we observed that SGP hybrid assemblies were completely devoid of both small (i.e. single base substitutions) and large assembly errors. Finally, we show the ability of SGP hybrid assembly to differentiate genomes of closely related bacterial isolates, suggesting its potential application in comparative genomics and pangenome analysis.

**Conclusion:**

Our results indicate the superiority of SGP hybrid assembly over both short-read and long-read assemblies with respect to completeness, contiguity, accuracy, and recovery of small replicons. By lowering the per-genome cost of sequencing, our parallel sequencing and hybrid assembly pipeline could serve as a cost effective and high throughput approach for completing high-quality bacterial genomes.

## Background

De novo genome assembly is a valuable tool for studying the biology of bacteria. From understanding the evolutionary processes underlying host adaptation [1] and development of drug resistance [2, 3], to investigating the genetic diversity among closely related bacteria [4, 5], to identification of novel biosynthetic gene clusters for discovery of therapeutically relevant natural products [6, 7], bacterial genomics research relies on accurate reconstruction of genomes from DNA sequencing reads. Currently, Illumina sequencing dominates the genomics field due to its low error rate and ever decreasing per-base cost of sequencing [8]. However, reads generated by Illumina platforms are typically shorter than repeat elements in bacterial genomes [9]. Consequently, de novo assembly using short reads often fails to resolve the majority of repeats in bacterial genomes, resulting in unfinished final assemblies composed of fragmented contiguous sequences (contigs) [10]. These draft genomes usually contain assembly errors that are problematic for accurate prediction of protein coding sequences (CDSs) and gene annotation [11].

On the other hand, single molecule sequencing technologies such as Pacific Biosciences (PacBio) and Oxford Nanopore Technologies generate sequencing reads of several kilobases, which can resolve the majority of repeat elements in bacterial genomes and improve the contiguity of assemblies. However, long reads generated by these platforms are error-prone [12], resulting in the introduction of single base substitutions and small insertions/deletions (indels) into the final assembly [13]. By taking advantage of both the accuracy of Illumina sequencing and the read length of single molecule sequencing, hybrid de novo assembly can resolve the majority of complex genomic structures (e.g. repetitive mobile elements) without compromising the accuracy of the final assembly [10, 13, 14]. The main limitation of this approach is its high per-genome cost of sequencing, particularly for preparing multiplexed (barcoded) long-read libraries, which can be limiting for large scale microbial genomics studies.

To address this limitation, we devised a methodological framework for hybrid sequencing and assembly of complete bacterial genomes without the need for multiplexing PacBio libraries. The driving idea behind our approach is that contigs generated by de novo assembly of barcoded short reads can be leveraged for sorting non-barcoded long reads of individual genomes within a moderately complex synthetic genomic pool. Subsequently, sorted long reads can be used to scaffold and resolve fragmented short-read assemblies via hybrid de novo assembly [10].

## Results

**Fig. 1:**
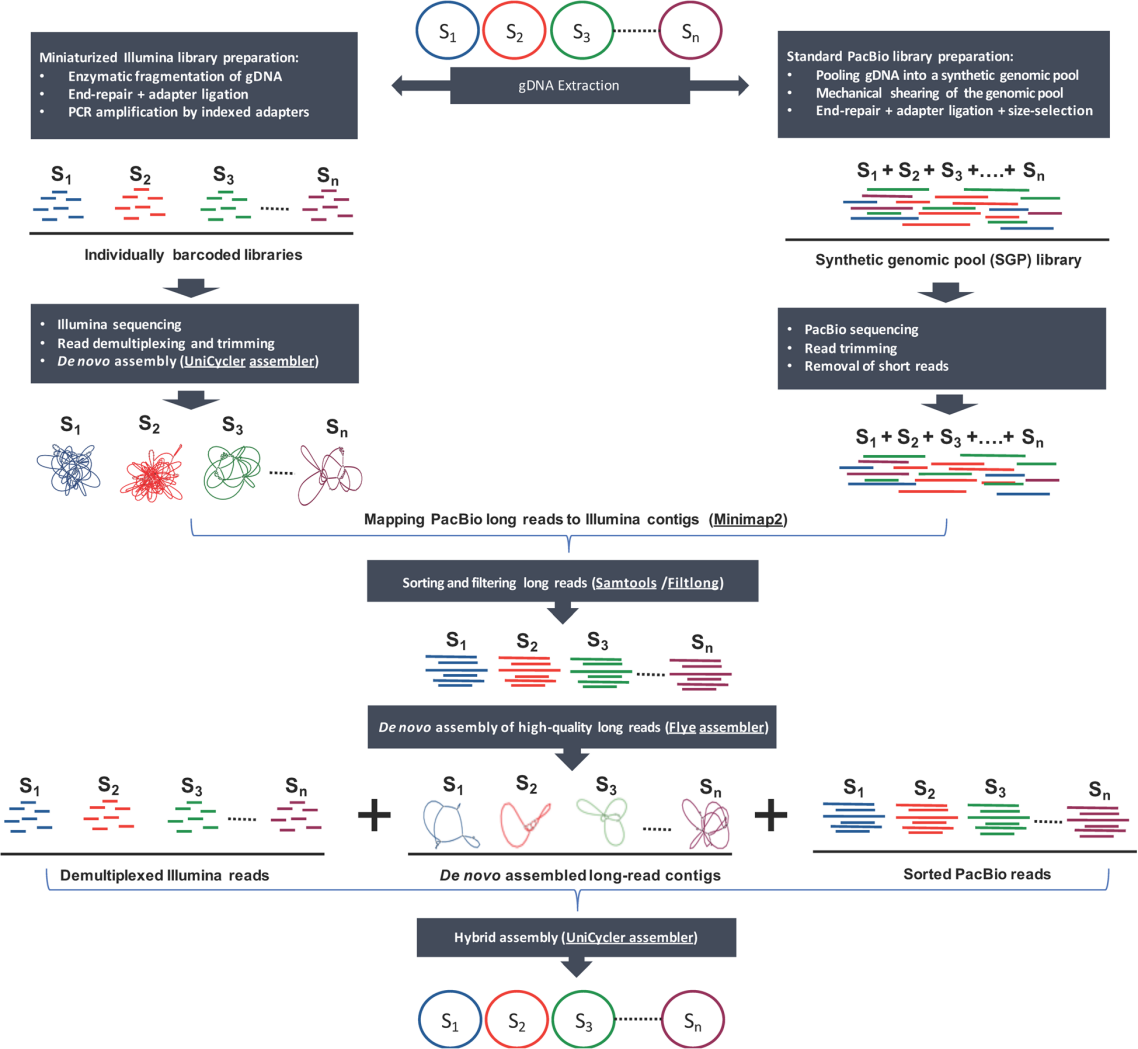
Schematic workflow for completion of draft bacterial genomes using long-read sequencing of synthetic genomic pools. Individual bacterial genomes are sequenced using a miniaturized, cost effective, multiplexed sequencing protocol on the Illumina platform. Short Illumina reads are used for de novo assembly of draft bacterial genomes (Illumina contigs). The gDNA of bacterial isolates are then combined into a synthetic genomic pool library (~80Mbp total genome size) and subjected to standard PacBio sequencing without multiplexing. Generated long reads are mapped to Illumina assemblies for sorting high-quality long reads of individual genomes. This is followed by *de novo* long-read assembly to generate ultra-long PacBio contigs for each genome, and finally, completion of draft bacterial genomes by hybrid assembly of Illumina short reads, PacBio long reads, and ultra-long PacBio contigs. The final assembly is polished by high-quality Illumina reads to correct potential assembly errors. Names of bioinformatics software used at each step of the assembly pipeline are indicated in parenthesis

**Fig. 2:**
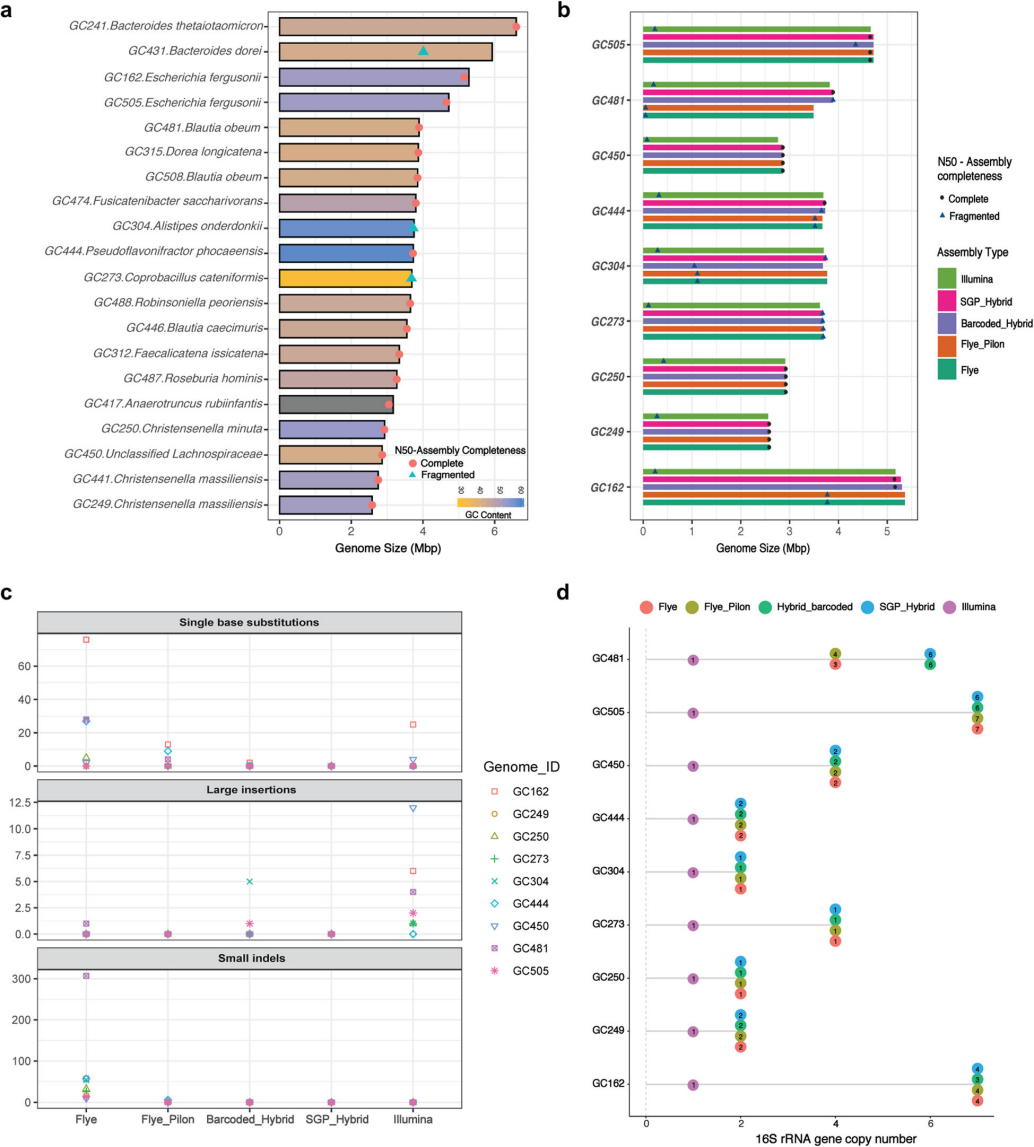
Overall performance and quality assessment of the SGP hybrid assembly pipeline. **a** Assembly statistics of 20 genomes subjected to the SGP hybrid assembly pipeline. The bar plot depicts genome size, GC content, N50 value, and assembly completeness of individual genomes (orange circles indicating complete assemblies and blue triangles indicating fragmented assemblies). **b** Evaluating the performance of SGP hybrid assembly by performing parallel multiplexed Illumina and PacBio sequencing on 9 genomes. The bar plot depicts assembly statistics of Illumina short-read assembly, hybrid assembly of barcoded Illumina and PacBio reads (Barcoded_Hybrid), SGP hybrid assembly, long-read assembly of barcoded PacBio reads polished using long reads (Flye) or short Illumina reads (Flye_Pilon). **c** Reference-free assembly validation: qualities of assemblies were assessed by mapping high-quality barcoded Illumina reads of each genome to its corresponding assemblies using Breseq. The top panel shows the frequency of single base substitutions, the middle panel shows the frequency of insertion sequences (unresolved repeat elements identified as small contigs with higher read coverages compared to other regions of the chromosome), and the bottom panel shows the frequency of small insertion-deletions (indels) in various assemblies of each genome. **d** Dot plot showing the ability of different assembly approaches to resolve multiple copies of the 16S ribosomal RNA gene within each genome. Position of each dot on x-axis depicts the total number of 16S rRNA gene copy numbers detected within each assembly. Numbers within dots indicate intragenomic heterogeneity (sequence variants) among 16S rRNA genes of each assembly

A schematic overview of the sequencing workflow and bioinformatics pipeline used for performing SGP hybrid assembly is provided in Fig. 1. We evaluated the precision of this protocol by sequencing the genomes of 20 isolates of the human gut microbiota, with different genome sizes (2.58–6.60 Mbp), GC contents (31.38–63.38%), and genomic similarity (Mash distances ranging from 0.00002–1.00; Additional file 1). By combining the genomic DNA of these isolates into a synthetic genomic pool (total size ~ 77 Mbp), we considerably reduced both the hands-on time and the cost of preparing long-read sequencing libraries compared to the standard PacBio multiplexing protocol (see Additional file 2 for a detailed comparison of the cost of SMRT library preparation between the SGP and standard multiplexing approach). Of the 20 genomes included in the SGP, we were able to assemble 17 complete genomes, 2 nearly complete ones (including *Alistipes onderdonkii* GC304, genome size = 3.75Mbp, N50 = 3.73Mbp, chromosomal contigs = 3; and *Coprobacillus cateniformis* GC273, genome size = 3.69Mbp, N50 = 3.68Mbp, chromosomal contigs = 3), and one partially fragmented genome (*Bacteroides dorei* GC431; genome size 5.93Mbp, N50 = 4.01Mbp, chromosomal contigs = 11, extrachromosomal circular assemblies = 7) (Fig. 2a and Additional file 3).

To evaluate the performance of the SGP hybrid assembly in the absence of reference genomes, we additionally performed standard PacBio multiplex sequencing on 9 out of 20 genomes included in the SGP. Gaining access to barcoded short and long reads of these genomes enabled us to comprehensively assess the quality of SGP hybrid assemblies in comparison with Illumina short-read assemblies, long-read-only assemblies polished either via PacBio reads (Flye assemblies) or Illumina reads (Flye_Pilon assemblies), and hybrid assemblies of barcoded Illumina and PacBio reads (barcoded hybrid assemblies). Among all assemblies tested, SGP hybrid assemblies were consistently the most contiguous, achieving N50 values equal to or greater than barcoded hybrid and long-read assemblies (Fig. 2b). With respect to long-read-only assemblies, we observed that contiguity and completeness of assembly could be greatly affected by read coverage. The Flye assembly of *Blautia obeum* GC481 was both shorter and more fragmented than its corresponding hybrid and Illumina assemblies. For this genome, the coverage of barcoded PacBio reads was considerably lower (~ 66x) than the other 8 genomes subjected to multiplexed PacBio sequencing (560 ± 244x; Additional file 4).

We further evaluated the ability of each assembly protocol to recover putative mobile elements (i.e. small replicons). For this purpose, the sequences of extrachromosomal circular assemblies detected by hybrid or long-read assemblies of each genome were aligned to different assemblies of that genome. Overall, SGP and barcoded hybrid assemblies performed best in the recovery of small replicons. Across all 20 genomes tested, SGP hybrid assembly recovered a total of 15 small replicons with high similarity to plasmid sequences in the NCBI nucleotide database (Additional file 5). Of these, 5 replicons belonged to the genomes that were subjected to multiplexed PacBio sequencing and were further confirmed by barcoded hybrid assembly. Illumina assembly was able to assemble 10 of the referred replicons whereas the sequences of the other 5 replicons were aligned to multiple chromosomal contigs in highly fragmented regions of the Illumina assemblies. On the other hand, Flye long-read assembly showed the poorest performance in recovery of plasmids, failing to assemble 3 replicons that were detected by both hybrid and Illumina assemblies (one belonging to *Christensenella minuta* GC250, and two belonging to *Pseudoflavonifractor phocaeensis* GC444).

We next performed reference-free assembly validation by mapping the high-quality Illumina reads of each genome to their corresponding assemblies using Breseq [15]. Here, we observed that SGP hybrid assemblies were completely devoid of errors such as single base substitutions, indels, or large insertions (Fig. 2c). In contrast, Flye assemblies polished by long reads contained the highest rate of single base substitutions and indels. Although polishing Flye assemblies via Illumina reads considerably reduced the frequency of small errors, particularly small indels, these assemblies still had a higher incidence of single base substitutions than corresponding hybrid assemblies. On the other hand, by mapping Illumina reads to Illumina assemblies, we identified a high frequency of small contigs, in the range of a few hundred bases, with higher read coverages compared to other regions of the chromosome. Alignment of these contigs to corresponding long-read and hybrid assemblies revealed multiple perfect matches throughout the genome, reflecting the inability of short-read assembly to resolve repeat elements. We further evaluated the ability of SGP hybrid assembly to resolve repeat elements by extracting and comparing the sequence and frequency of 16S rRNA genes among different assemblies of individual genomes. Our results indicated that SGP assemblies, along with other long-read and hybrid assemblies, were able to resolve multiple copies of the 16S rRNA gene for each genome (ranging between 2 and 7), whereas Illumina assemblies only contained a single copy of this gene (Fig. 2d). In addition, hybrid and long-read assemblies were able to detect varying levels of intragenomic heterogeneity in 16S rRNA genes that would otherwise go undetected by Illumina assembly (Fig. 2d and Additional file 6).

We also performed whole genome alignments between the barcoded hybrid assembly of each isolate as the reference genome and all other assemblies of that isolate using nucmer [16]. We observed that with the exception of two genomes, GC162 and GC304, SGP assemblies contained no single nucleotide variations (SNVs) in relation to their corresponding barcoded hybrid assemblies (Additional file 7). For GC304, the barcoded hybrid assembly was more fragmented (29 contigs, N50 = 1.05Mbp) than its corresponding SGP hybrid assembly (2 contigs, N50 = 3.7Mbp), and therefore not suitable to serve as the reference genome for whole genome alignment. However, for GC162, both barcoded and SGP hybrid assemblies were complete, and comprised one chromosomal contig and one plasmid. For this genome, SGP hybrid assembly contained a number of SNVs, including 148 base substitutions, 5 insertions, and 12 deletions in comparison with barcoded hybrid assembly. We also observed a size difference between the chromosomal contigs of the two assemblies (5,295,203 bp for barcoded hybrid assembly and 5,282,480 bp for SGP hybrid assembly). This was further investigated by the “show-diff” function of the nucmer package to investigate potential large gaps/duplications between the two assemblies. Here we were able to detect a large duplication event in the barcoded hybrid assembly between the sequence coordinates 2,003,437 - 2,021,745. By closely examining the results of the breseq analysis, we observed a “missing coverage” event spanning the same sequence coordinates of the barcoded hybrid assembly (1,994,402 – 2,039,909), suggesting the presence of a potential false duplicate in the assembly, whereas breseq analysis did not identify any missing coverage for the SGP hybrid assembly of GC162.

**Fig. 3:**
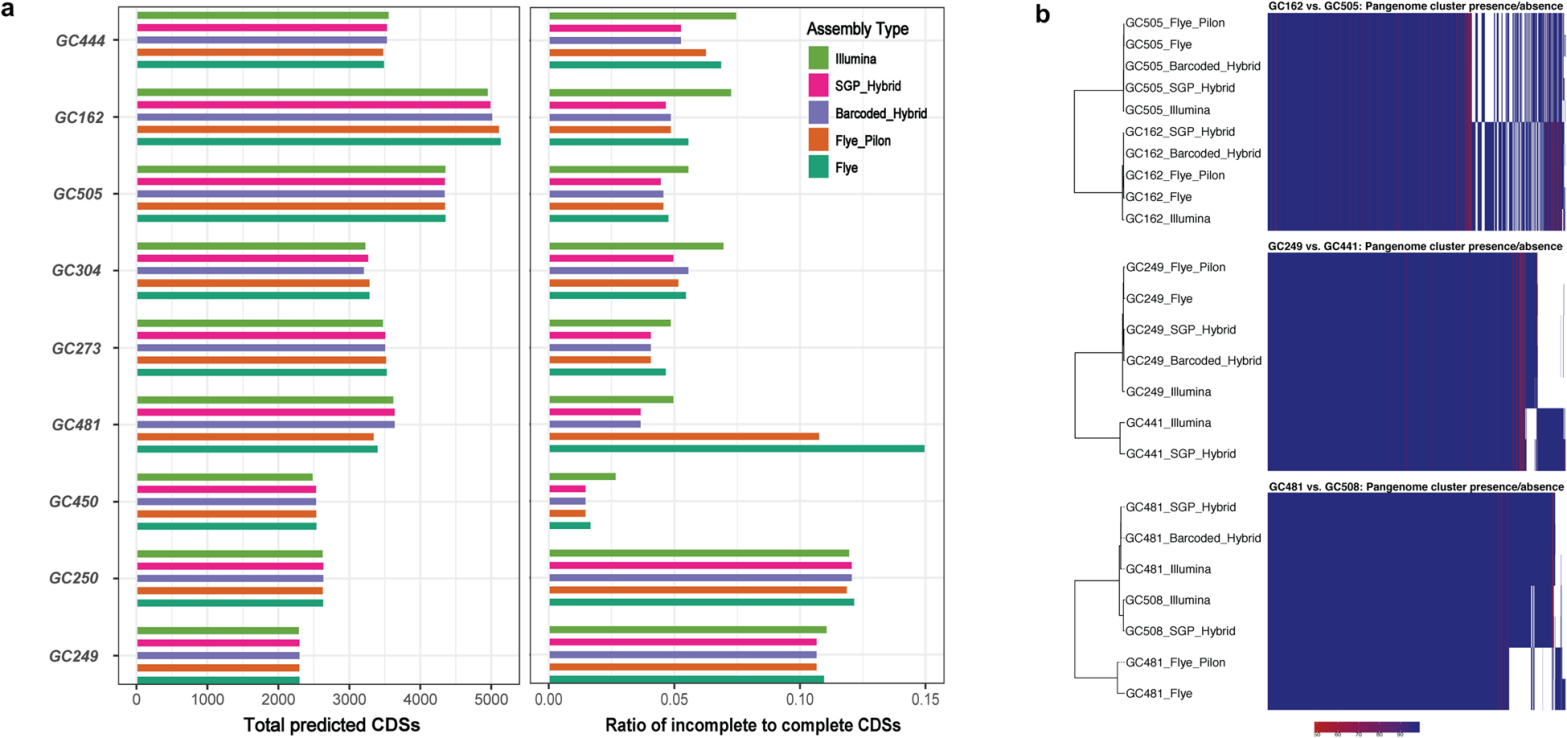
Evaluating the effect of assembly quality on gene annotation. **a** Hybrid assembly improves gene prediction and annotation accuracy. The left bar plot depicts total number of predicted coding sequences (CDSs) of various assemblies of each genome. The bar plot on the right shows the ratio of incomplete to complete CDSs of each assembly. CDSs were predicted by Prodigal and aligned to the UniProtKB/TrEMBL protein database using DIAMOND Blastp. The ratio of query sequence length to subject sequence length was then used as a proxy to measure completeness of the predicted CDSs (threshold of ≥0.95). **b** Pangenome analysis of different assemblies of related strains, including *Escherichia fergusonii* (GC162 vs. GC505), *Christensenella massiliensis* (GC249 vs. GC441) and *Blautia obeum* (GC481 vs. GC508). Phylogenetic trees were generated by alignment of core and accessory genes identified by PIRATE. The colour ramp indicates the Markov clustering (MCL) threshold at which each gene family has been classified (the higher this threshold, the less divergent is that gene family across assemblies)

To assess the impact of assembly contiguity and completeness on gene annotation, we next aligned the predicted CDSs of each assembly to the UniProtKB/TrEMBL protein database [17]. Overall, the numbers of predicted CDSs were roughly comparable among different assemblies of each genome. However, by comparing the length of predicted CDSs to protein sequences in the reference database, we observed that completeness of predicted CDSs was greatly affected by assembly contiguity (Fig. 3a). Overall, SGP hybrid assemblies yielded the most accurate CDS predictions, whereas Illumina assemblies contained the highest ratio of incomplete CDSs. Regarding long-read assemblies, we observed that unpolished Flye assemblies yielded higher proportions of incomplete CDSs than Flye_Pilon assemblies, reflecting the high frequency of small errors such as single base substitutions and indels in these assemblies. Lastly, to assess the potential application of the SGP approach for sequencing and assembly of closely related bacteria, we performed pangenome analyses [18] on different assemblies of 3 pairs of related strains, including *Escherichia fergusonii* strains GC162 and GC505, *Christensenella massiliensis* strains GC249 and GC444, and *Blautia obeum* strains GC481 and GC508. Here, we observed that each SGP assembly consistently clustered together with the Illumina assembly and, when available, barcoded hybrid assembly of the same genome rather than related isolates (Fig. 3b).

## Discussion

The vast majority of bacterial genomes deposited in public databases have been generated using short-read sequencing platforms, and are therefore of draft quality and incomplete. Recent developments in single molecule sequencing technologies have enabled assembly of complete bacterial genomes [19–21]. However, despite efforts to improve the accuracy of long-read de novo assembly algorithms [22–25], the high error rate of reads generated by PacBio and Oxford Nanopore platforms can result in the introduction of assembly errors [13]. In addition, the high per-genome cost of library preparation, particularly for multiplexed PacBio sequencing, is a limiting factor for widespread application of long-read sequencing in bacterial genomics. In this study, we devised a high-throughput protocol for finishing draft bacterial genomes using non-barcoded PacBio sequencing of synthetic genomic pools. This, together with the use of our miniaturized Illumina library preparation protocol, provides a cost-effective approach for parallel sequencing and hybrid assembly of complete bacterial genomes. The SGP library used in this study was composed of 20 bacterial genomes and sequenced on a single PacBio SMRT cell. However, the average long-read coverage achieved for individual genomes was considerably higher than what is required for hybrid de novo assembly, indicating a higher throughput potential of our approach.

Our results indicate that hybrid assemblies have improved contiguity compared to both short- and long-read-only assemblies, and for some genomes, SGP hybrid assemblies were even more contiguous than corresponding barcoded hybrid assemblies. Improved contiguity of SGP assemblies can be attributed to the ability of this approach to overcome the size restriction of barcoded PacBio libraries. In multiplexed sequencing, successful demultiplexing of reads relies on the passage of barcodes, ligated to both ends of the reads, through immobilized polymerases at the bottom of zero-mode waveguide (ZMW) units of PacBio SMRT cells [26]. This requirement limits both the insert size and read length of barcoded libraries. By precluding the need for barcoding individual libraries, our SGP library preparation overcame the insert size limitation and yielded reads that were on average longer than barcoded reads (~ 12Kbp vs. 8Kbp, respectively; Additional file 4). Contiguity and completeness of long-read-only assembly can also be greatly affected by read coverage [22, 24]. As indicated, Flye assemblies of *Blautia obeum* GC481 were shorter and less contiguous than both SGP and barcoded hybrid assemblies. Improved contiguity and completeness of barcoded hybrid assembly of this genome indicate the advantage of hybrid assembly in resolving genomic structures using lower coverages of long reads [10].

Another limitation of long-read sequencing and assembly is the possibility of excluding small replicons, e.g. small plasmids and phages, during library preparation. The current size-selection threshold of 10-15Kbp recommended for multiplexed PacBio sequencing can lead to unwitting depletion of small plasmid DNA molecules from the final sequencing libraries. In the present study, Illumina and hybrid assemblies of *Christensenella minuta* GC250 and *Pseudoflavonifractor phocaeensis* GC444 contained plasmid sequences, in the range of 5-6Kbp, that did not match any region of the Flye assemblies of these genomes. As mobile elements play critical roles in evolution of bacteria, including acquisition of antimicrobial resistance via plasmids [27] and transfer of virulence genes via phages [28], failure to reliably assemble small replicons can greatly limit the application of long-read-only assembly in bacterial genomics. On the other hand, we observed that short-read-only assembly fails to resolve repeat elements in bacterial genomes. Many of the repeat regions in bacterial genomes could represent mobile genetic elements such as bacteriophages, conjugative transposons, and insertion sequences, which can all contribute to pangenome evolution and accessory gene acquisition [29–31]. The 16S rRNA gene also serves as a good example of a multi-copy gene in the majority of bacterial genomes [32]. The number of rRNA gene copies in a bacterial genome could be reflective of the ecological strategies of that organism to respond to changing environments and nutrient availability [33]. In addition, obtaining a better understanding of rRNA gene copy number across phylogenetically diverse bacterial genomes could help overcome the biases of amplicon profiling of complex microbial communities [34]. In the present study, hybrid and long-read assemblies of the majority of genomes contained multiple copies of the rRNA gene, whereas Illumina assemblies failed to capture this important intragenomic heterogeneity.

Contiguity and completeness of assembly also have critical impacts on the accuracy of gene prediction, and consequently, gene annotation [35, 36]. In this study, Illumina assemblies contained the highest ratio of incomplete CDSs, reflecting the presence of partial open reading frames (ORFs) in fragmented contigs of short-read assemblies [35]. Besides assembly fragmentation, small errors such as indels and base substitutions can cause frameshifts in ORFs and increase the frequency of erroneous pseudogenes. Having the highest frequency of single base substitutions and indels, unpolished Flye assemblies yielded higher proportions of incomplete CDSs than Flye_Pilon assemblies, indicating the limitation of long-read-only assembly for accurate gene annotation.

Despite demonstrated advantages of SGP hybrid assembly over both long- and short-read-only assemblies, there are certain caveats to consider with our results. First, although our preliminary assessment indicates that SGP hybrid assembly can accurately resolve draft genomes of closely related strains, further application of this approach in large-scale epidemiological studies and pangenome analysis of closely related strains, in particular those with more complex genomic structures (e.g. high frequency of repetitive mobile elements), remains to be experimentally validated. Second, our study did not assess the throughput of the SGP hybrid assembly approach, i.e. determine the upper size limit of the SGP to achieve optimal resolution of draft bacterial genomes. However, with advances in PacBio SMRT sequencing technology and introduction of new platforms that generate a considerably higher number of reads per SMRT cell, it may be possible to push the upper size limit of a single SGP library without sacrificing per-genome coverage of the long reads.

## Conclusions

Due to the popularity of Illumina sequencing, thousands of bacterial genomes have already been sequenced using this technology worldwide. However, incompleteness of draft genomes generated by Illumina reads limits their application for downstream genomics analyses. With single molecule sequencing technologies becoming increasingly available, long-read assembly is gaining popularity among microbiologists. Here, we demonstrated the limitations of both short-read and long-read assemblies for bacterial genomics, and showed the overall superiority of the hybrid assembly with respect to completeness, contiguity, accuracy, and recovery of small replicons. By decreasing the per-genome cost of sequencing and overcoming the size restriction of barcoded long-read assembly, we showed that SGP hybrid sequencing and assembly is a cost effective and high throughput approach for completing bacterial genomes.

## Methods

### Media and culture conditions for isolation of human gut strains

Bacteria used in this study were isolated as part of a comprehensive culturomics screening of the human gut microbiota conducted at McMaster University (Hamilton, ON, Canada), a project approved by the Hamilton Integrated Research Ethics Board. In brief, fresh fecal samples were donated by healthy individuals with no gastrointestinal symptoms and no history of antibiotic therapy within 3 months of collection. Immediately after defecation, fecal samples were transferred to a sterile container and stored in sealed bags containing an anaerobic pouch (GasPak™ EZ; BD, Sparks, MD, USA) and ice-pack. Samples were transferred to the laboratory within 3 h of collection and were further processed in an anaerobic chamber (5% CO2, 5% H2, 90% N2; Shel Labs, Cornelius, OR, USA).

The following media were used to culture and isolate the original strains: a) brain heart infusion (BHI) agar (BD) supplemented with 0.5 g/L L-cysteine hydrochloride hydrate, 10 mg/L hemin, and 1 mg/L vitamin K for isolation of GC241:*Bacteroides thetaiotaomicron,* GC273:*Coprobacillus cateniformis,* GC304:*Alistipes onderdonkii,* GC312:*Faecalicatena fissicatena,* and GC315:*Dorea longicatena,* b) 0.2× BHI + 1.5% (w/v) agar (BD) supplemented with 0.5 g/L pectin for isolation of GC481:*Blautia obeum*, GC487:*Roseburia hominis,* and GC488:*Robinsoniella peoriensis,* c) Cooked Meat Broth + 1.5% (w/v) agar (BD) for isolation of GC446:*Blautia caecimuris* and GC508:*B. obeum*, d) Fastidious anaerobe agar (Neogen, Lansing, MI, USA) for isolation of GC417:*Anaerotruncus rubiinfantis*, GC444:*Pseudoflavonifractor phocaeensis,* and GC474:*Fusicatenibacter saccharivorans,* e) Gifu anaerobic medium (Himedia Laboratories, Mumbai, India) for isolation of GC249 and GC441:*Christensenella massiliensis*, GC250:*Christensenella minuta*, GC431:*Bacteroides dorei*, and GC450:*Unclassified Lachnospiraceae*, and f) MacConkey agar (BD) for isolation of GC162 and GC505:*Escherichia fergusonii*.

### Library preparation and sequencing

Genomic DNA (gDNA) of all strains was extracted using the Wizard Genomic DNA Purification Kit (Promega, Madison, WI, USA). Pulsed field gel electrophoresis (CHEF-Mapper XA; Bio-Rad, Hercules, CA, USA) was used to assess the quality of extracted gDNA and availability of large DNA fragments (>25kbp; required for generation of long-read PacBio libraries). DNA concentration was measured by Qubit dsDNA HS kit (ThermoFisher Scientific, Mississauga, ON, Canada).

Illumina short-read libraries (*n* = 20) were prepared according to a miniaturized library preparation protocol (Additional file 8) using the NEBNext Ultra II FS DNA Library Prep Kit for Illumina (NEB, Ipswich, MA, USA). Individually barcoded libraries were subjected to dual size-selection using the ProNex® Size-Selective Purification System (Promega, Madison, WI, USA) to enrich for insert sizes of 800-1000 bp. Final libraries were sequenced on an Illumina HiSeq2500 platform in rapid run mode, paired-end 2x250nt, at the McMaster Metagenomics Facility (Hamilton, ON, Canada).

PacBio Single Molecule Real-Time (SMRT; Pacific Biosciences, Menlo Park, CA, USA) libraries were prepared using either the standard multiplexing protocol or a non-barcoded approach used for generation of SGP libraries. For multiplexed (i.e. barcoded) libraries, 2 μg of gDNA from each strain (*n* = 9; including GC162, GC249, GC250, GC273, GC304, GC444, GC450, GC481, GC505) was diluted in 200 μL of elution buffer and fragmented using g-TUBE (Covaris, Woburn, MA, USA) by centrifuging at 2400×g for 3 min. Fragmented gDNA was concentrated using AMpure PB magnetic beads (Beckman Coulter, Brea, CA, USA) and used for preparation of libraries following the manufacturer’s protocol for multiplexed 10 kb SMRTbell Express Template Prep Kit 2.0 (Pacific Biosciences). Individually barcoded libraries were subjected to BluePippin size-selection (Sage Science, Inc., Beverly, MA, USA) for enrichment of SMRTbell templates greater than 10 kb. Final libraries were sequenced using a SMRT Cell on the PacBio Sequel system at the McMaster Genome Facility. In the non-barcoded approach, a synthetic genomic pool was generated by combining equal amount of gDNA (200 ng) from 20 human gut isolates, including the above mentioned 9 strains used for preparation of the multiplexed libraries, plus an extra 11 strains selected based on genome size, GC content, and phylogentic diversity (GC241, GC312, GC315, GC417, GC431, GC446, GC474, GC487, GC488, and GC508). 3 μg DNA of the resulting SGP was diluted in 200 μL of elution buffer and fragmented using g-TUBE (Covaris) by centrifuging at 1900×g for 3 min. The fragmented DNA was concentrated using AMpure PB magnetic beads (Beckman Coulter) and used for preparation of a single non-multiplexed sequencing library following the manufacturer’s protocol for 20 kb SMRTbell Express Template Prep Kit 2.0 (Pacific Biosciences) size selected by BluePippin to enrich for SMRTbell templates greater than 10 kb. The final library was sequenced using a SMRT Cell on the PacBio Sequel system at the McMaster Metagenomics Facility.

### De novo assembly of short and long reads

The following approaches were used for assembly of short and long reads (detailed descriptions of required bioinformatics packages, command lines and parameters used for each step of the bioinformatics pipeline is provided in Additional file 9):

- Assembly of barcoded short reads: Trimmomatic (v.0.39) [37] was used for quality trimming and removal of adapters from Illumina reads. Quality of resulting sequences were assessed by FastQC (v.0.11.7) [38]. Unicycler (v.0.4.8) [10] was then used for de novo assembly of Illumina reads (Illumina assemblies; *n* = 20), followed by exhaustive Pilon (v.1.23) [39] polishing to correct assembly errors such as base substitutions, insertions, and deletions, until no further changes were observed.
- Assembly of barcoded long reads: PacBio subreads were extracted for individually barcoded libraries and assembled by Flye (v.2.5) [22] using default parameters (−-plasmids --iterations 1 --genome-size = “estimated based on Illumina assemblies”). The resulting assemblies were used for downstream analyses either without further polishing (Flye assemblies; *n* = 9), or after 5 rounds of polishing by Pilon, using Illumina short reads (Flye_Pilon assemblies; n = 9).
- Hybrid assembly of barcoded libraries: Unicycler was used for hybrid assembly of barcoded short and long reads from each strain (Barcoded Hybrid assemblies; n = 9). By default, Unicycler performs a SPAdes [40] assembly of the Illumina reads to create a set of high-quality anchor contigs. It then uses Minimap and Miniasm [23] for overlapping and de novo assembly of long reads which are further used for scaffolding the assembly and bridging the gaps between Illumina anchor contigs.
- Hybrid assembly of barcoded short reads and SGP: In order to demultiplex SGP into individual genomes, SGP long reads were mapped to Illumina assemblies using minimap2 [41] with parameters set for mapping PacBio reads to reference genomes (−ax map-pb). Samtools (v.1.7) [42] was used for extraction of the long reads that were mapped to each of the Illumina assemblies. Extracted long reads for each genome were then filtered and trimmed by Filtlong (v.0.2.0, https://github.com/rrwick/Filtlong). Parameters used for Filtlong filtering and trimming of long reads included: length (minimum 2 kb) and their quality as determined by matching k-mers to corresponding Illumina reads of each isolate used as external references. When necessary, long reads were trimmed by removing bases from the start and end of the read which did not match a k-mer in the reference Illumina reads, or by splitting reads whenever 100 consequent bases failed to match a k-mer in the Illumina reads. For each genome, 90% percent (or a maximum of 500 Mbp) of the highest quality long reads were retained. In the next step, selected long reads from each genome were combined with corresponding high-quality contigs from Illumina assemblies, and together were subjected to Flye long-read assembly. Lastly, the combination of original Illumina short reads, high-quality long reads selected by Filtlong, and abovementioned Flye long-read assemblies were used to perform hybrid assembly by Unicycler (SGP Hybrid assemblies; *n* = 20). Here, Flye long-read contigs replaced the default long-read assembly pipeline of the Unicycler (Minimap and Miniasm) to scaffold the assembly, and when possible, selected high-quality long reads were used to directly bridge remaining gaps between Illumina anchor contigs. The final assembly was subjected to exhaustive Pilon polishing using original Illumina reads to correct potential assembly errors introduced by long reads, until no further changes were observed.

### Comparison of the quality of assemblies

Mash (v.2.1.1) [43] was used for measuring the genomic distances of all isolates and building the distance tree. Quast (v.5.0.2) [44] was used to determine genome size, GC content, and contiguity (N50) of individual assemblies. In order to evaluate the frequency of common assembly errors such as base substitutions, small insertions and deletions (indels), and large deletions, Breseq (v.0.34.0) [15] was used to map high-quality Illumina reads from each genome to its corresponding individual assemblies. For the 9 genomes that were subjected to multiplexed PacBio sequencing, assembly qualities were further assessed using Nucleotide MUMmer (nucmer; v.3.1) [16] by aligning each assembly to its corresponding barcoded hybrid assembly as a “reference genome” of that isolate. In order to evaluate the effect of each assembly approach on gene prediction and annotation, Prodigal (v.2.6.3) [45] was used to predict protein-coding sequences (CDSs) of each assembly. The amino acid sequences of identified CDSs were aligned to UniProtKB/TrEMBL protein database [17] using DIAMOND Blastp (v.0.8.36) [46]. The ratio of query sequence length to subject sequence length was then used as a proxy to measure completeness of the predicted CDSs (threshold of ≥0.95). Furthermore, PIRATE (v.1.0.3) [18] pangenome analysis was used for comparison of CDS presence/absence and the performance of the SGP hybrid assembly in differentiating between closely related strains within the same species. The 16S rRNA genes within each assembly were identified and extracted using Barrnap version 0.9 (https://github.com/tseemann/barrnap).

### Assessment of extrachromosomal circular assemblies

The ability of different assembly protocols to recover mobile elements was assessed by screening extrachromosomal circular assemblies for the presence of plasmids and phages. Bandage (v.0.8.1) [47] was used to detect and extract the sequences of all small and large extrachromosomal circular assemblies associated with each genome. The sequences of these assemblies were screened for the presence of potential plasmids and phages by mapping against the NCBI nucleotide database using blastn [48].

## Supplementary information

**Additional file 1.** Genomic distances of selected human gut isolates. Mash (v.2.1.1) was used for measuring the genimic distances of all isolates and b uilding the distance matrix. The scale bar depicts a Mash distance of 1.0 (A MASH distance of 0.05 corresponds to ~ANI of 95%, or ~ 70% DNA-DNA reassociation, a historical approximation for bacterial species definition [Ondov et al. 2016]”.

**Additional file 2.** Comparison of the cost of PacBio SMRT library preparation between standard multiplexing approach and SGP

**Additional file 3. ** Quast summary statistics of assembly qulaities.

**Additional file 4.** Length and genome coverage of PacBio long reads for both SGP and barcoded hybrid assemblies.

**Additional file 5.** Blast results of extrachromosomal replicons against NCBI nucleotide database.

**Additional file 6.** Intragenomic heterogeneity in 16S rRNA genes.

**Additional file 7.** Reference-based assessment of the accuracy of different assemblies. The accuracy of individual assemblies of each isolate were assessed by performing whole genome alignment between the barcoded hybrid assembly of each isolate as the reference genome and all other assemblies of that isolate using nucmer. The top panel shows the frequency of single base substitutions, the middle panel shows the frequency of single nucleotide deletions and the bottom panel shows the frequency of single nucleotide insertions in various assemblies of each isolate in relation to its barcoded hybrid assembly.

**Additional file 8.** Miniaturized shotgun library preparation protocol for Illumina sequencing

**Additional file 9.** Detailed description of required bioinformatics packages, command lines and parameters used for each step of the bioinformatics pipeline.

## Data Availability

The sequencing data were deposited into the Sequence Read Archive (SRA) of NCBI (http://www.ncbi.nlm.nih.gov/sra) and can be accessed via BioProjects number PRJNA603771 for barcoded Illumina and PacBio reads and PRJNA603756 for SGP PacBio reads. Bioinformatics pipeline for reproducing the results of this manuscript is provided as bash scripts available in Additional file 9.

## Abbreviations

SGP: Synthetic genomic pool
CDS: Protein coding sequence
SMRT: Single molecule real-time
