# Supplementary material for: Completion of draft bacterial genomes by long-read sequencing of synthetic genomic pools
Source: BMC Genomics. 2020 Jul 29;21:519. doi: 10.1186/s12864-020-06910-6 (PMC7392658; doi:10.1186/s12864-020-06910-6)
Supplement: Supplementary file 1 — Additional file 1. Genomic distances of selected human gut isolates. Mash (v.2.1.1) was used for measuring the genimic distances of all isolates and b uilding the distance matrix. The scale bar depicts a Mash distance of 1.0 (A MASH distance of 0.05 corresponds to ~ANI of 95%, or ~ 70% DNA-DNA reassociation, a historical approximation for bacterial species definition [Ondov et al. 2016]”. [file 12864_2020_6910_MOESM1_ESM.pdf]

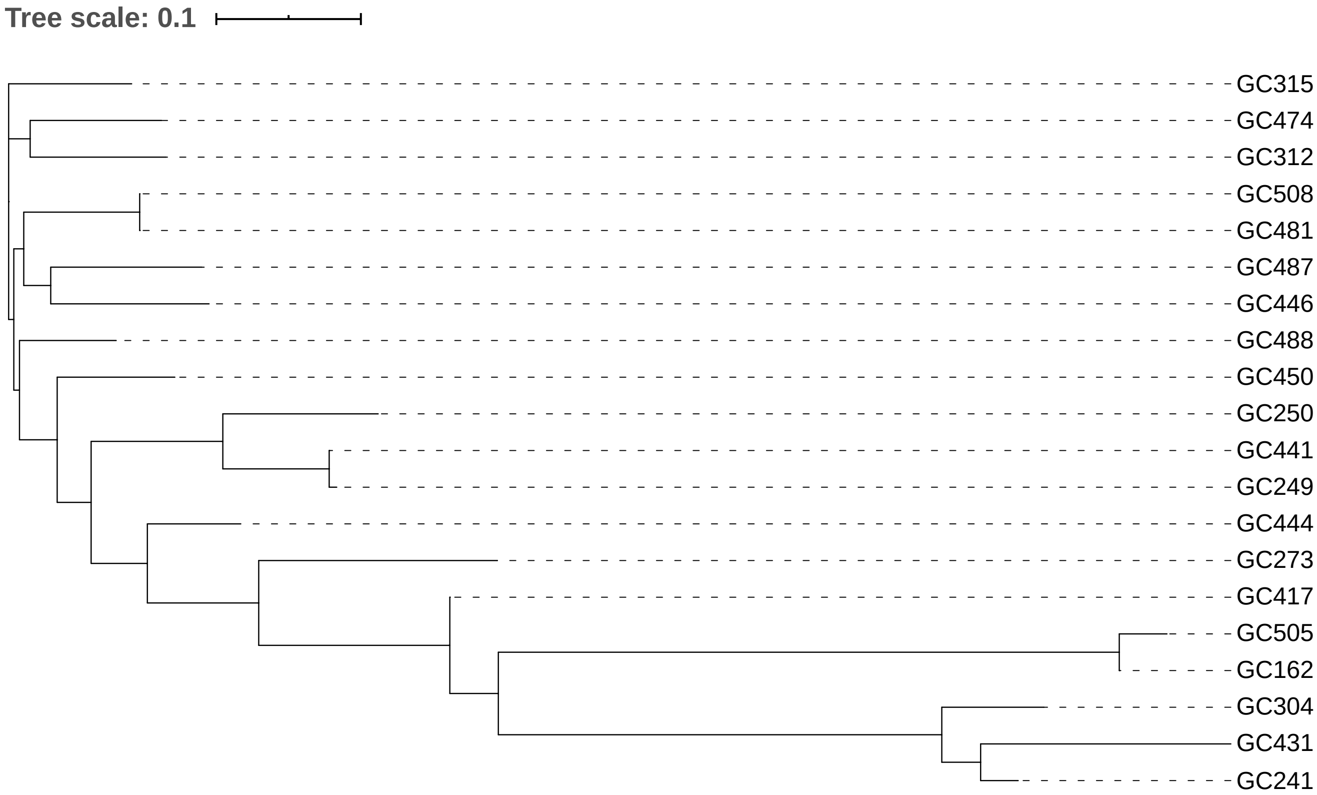

Additional File.1. Genomic distances of selected human gut isolates. Mash (v.2.1.1) was used for measuring the genomic distances of all isolates and building the distance matrix. The scale bar depicts a Mash distance of 0.1 (A MASH distance of 0.05 corresponds to ~ANI of 95%, or ~70% DNA-DNA reassociation, a historical approximation for bacterial species definition [Ondov et al. 2016] ”.
