# Supplementary material for: Completion of draft bacterial genomes by long-read sequencing of synthetic genomic pools
Source: BMC Genomics. 2020 Jul 29;21:519. doi: 10.1186/s12864-020-06910-6 (PMC7392658; doi:10.1186/s12864-020-06910-6)
Supplement: Supplementary file 2 — Additional file 2. Comparison of the cost of PacBio SMRT library preparation between standard multiplexing approach and SGP [file 12864_2020_6910_MOESM2_ESM.docx]

| **Additional File.2.** Comparison of the cost of PacBio SMRT library preparation between standard multiplexing approach and SGP | | | | | |
| --- | --- | --- | --- | --- | --- |
|  | **Library preparation approach** | | | | |
|  | **Standard Multiplexing** | | | **Synthetic Genomic Pool (SGP)** | |
| Reagent/Kit | **Required** | **Cost per genome (CAD)** | **Cost per 11 genomes^1^** | **Required** | **Cost per 20 genomes (CAD)** |
| SMRTbell Express Template Prep Kit 2.0 | Yes | $72 | $792 | Yes | $72 |
| AMPure PB beads | Yes | ~$15 | $165 | Yes | $15 |
| G-Tube | Yes | $49 | $539 | Yes | $49 |
| Barcoded Overhang Adapter | Yes | $10 | $110 | No | NA |
| BluePippin™ Size Selection | Yes | ~$73 | $73 | Yes | $73 |
| QC (Qubit, TapeStation, etc.) and laboratory consumables | Yes | $25 | $275 | Yes | $25 |
| Total cost^2^ |  | **$244** | **$1954** |  | **$234** |
| ^1^A subset of 11 bacterial genomes, out of 20 genomes subjected to (SGP), were selected and subjected to standard multiplexing PacBio library preparation and sequencing on a single SMRT PacBio cell. The reason for including fewer number of genomes in the standard multiplexing approach was to achieve adequate sequencing depth for performing long-read only assembly. Out of 11 genomes included in the standard multiplexing approach, only 9 yielded adequate sequencing depth to be included in downstream analyses.  ^2^In addition to the cost-savings achieved through SGP library preparation and sequencing approach, our miniaturized library preparation explained in the Additional File.8 also results in significant cost-savings over the standard Illumina library preparation protocol (~12CAD per genomes vs. ~50CAD per genome, using the NEB-Next UltraII FS DNA library prep kit). | | | | | |
|  | | | | | |
