# Supplementary material for: Completion of draft bacterial genomes by long-read sequencing of synthetic genomic pools
Source: BMC Genomics. 2020 Jul 29;21:519. doi: 10.1186/s12864-020-06910-6 (PMC7392658; doi:10.1186/s12864-020-06910-6)
Supplement: Supplementary file 7 — Additional file 7. Reference-based assessment of the accuracy of different assemblies. The accuracy of individual assemblies of each isolate were assessed by performing whole genome alignment between the barcoded hybrid assembly of each isolate as the reference genome and all other assemblies of that isolate using nucmer. The top panel shows the frequency of single base substitutions, the middle panel shows the frequency of single nucleotide deletions and the bottom panel shows the frequency of single nucleotide insertions in various assemblies of each isolate in relation to its barcoded hybrid assembly. [file 12864_2020_6910_MOESM7_ESM.pdf]

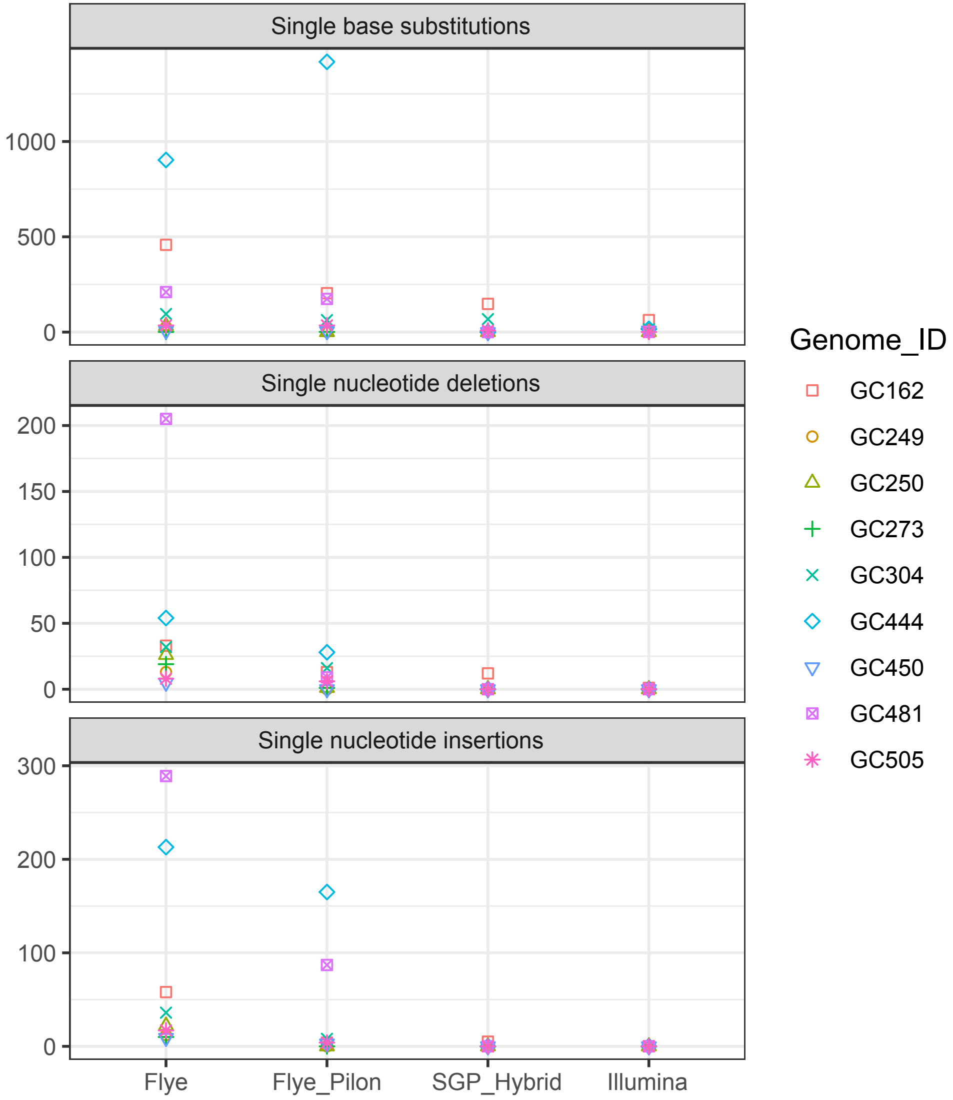

Additional File.7. Reference-based assessment of the accuracy of different assemblies. The accuracy of individual assemblies of each isolate were assessed by performing whole genome alignment between the barcoded hybrid assembly of each isolate as the reference genome and all other assemblies of that isolate using nucmer. The top panel shows the frequency of single base substitutions, the middle panel shows the frequency of single nucleotide deletions and the bottom panel shows the frequency of single nucleotide insertions in various assemblies of each isolate in relation to its barcoded hybrid assembly.
