# Supplementary material for: Completion of draft bacterial genomes by long-read sequencing of synthetic genomic pools
Source: BMC Genomics. 2020 Jul 29;21:519. doi: 10.1186/s12864-020-06910-6 (PMC7392658; doi:10.1186/s12864-020-06910-6)
Supplement: Supplementary file 8 — Additional file 8. Miniaturized shotgun library preparation protocol for Illumina sequencing [file 12864_2020_6910_MOESM8_ESM.docx]

**Miniaturized shotgun library preparation protocol for Illumina sequencing**

**This is a modified protocol for use with NEB-Next UltraII FS DNA library prep (Catalog# E7805L available @** [**https://international.neb.com/products/e7805-nebnext-ultra-ii-fs-dna-library-prep-kit-for-illumina#Product%20Information)**](https://international.neb.com/products/e7805-nebnext-ultra-ii-fs-dna-library-prep-kit-for-illumina#Product%20Information))

Following reagents/material are not included in the kit and should be ordered separately:

- USER enzyme from NEB (catalog# M5505L)
- NEB Adapter and Indexed barcodes (i5 and i7 indexes) can be ordered from NEB (catalog# E7600 and E7780) or alternatively ordered form oligo providers such as IDT
- NEBNext® Ultra II Q5® Master (in addition to what provided in the kit, catalog# M0544L)
- Pronext size selection system from Promega (“https://www.promega.ca”catalog number NG2002): required for purification and size selection of libraries.
- Magnetic stand for bead-purification from any provider

Pre-processing and technical notes:

- Input DNA should be normalized to a constant concentration across samples (e.g. 2-5ng/uL using Qubit) to ensure a more or less similar library yield using limited number of PCR cycles (7 cycles). If DNA input is below 2ng/uL, then use 8 cycles of PCR
- Reagents should be thawed on ice and pipette mix thoroughly before use
- Dilute NEB-next adaptor on 1:1 volume with water or preferably 10 mM Tris-HCl, pH 7.5 +10mM NaCl
- When using multichannel pipette and working on 96-well plate, calculate 10% additional reagents for residues and pipetting error. The remainders can be kept at -20 and use for future lib preps.

The followings are the reagent volume and reaction conditions used at each step:

- Step1) DNA fragmentation and end repair

|  | x1 |
| --- | --- |
| gDNA | 6.5uL |
| UltraII FS buffer | 1.75uL |
| UltraII FS enzyme mix | 0.5uL |
|  | 8.75uL |

- Buffer and enzyme can be mixed and use as a mastermix to reduce pipetting error
- Incubate the mixture in a thermal cycler with following cycles:
- 3:45min@37°C
- 30min@65°C
- hold@4°C
- Lid heated@75°C
- Step2) Adaptor ligation

|  | x1 |
| --- | --- |
| Fragmented DNA from previous step | 8.75uL |
| UltraII ligation MM | 7.5uL |
| UltraII ligation enhancer | 0.25uL |
| NEB adaptor | 0.63uL |

- Ligation MM and enhancer can be mixed and use as a mastermix to reduce pipetting error, but adapter should be added separately to avoid adapter-dimer formation
- Incubate the mixture in a thermal cycler with following cycles:
- 15min@20°C
- Lid not heated
- Step3)
- Premix USER enzyme and Cutsmart buffer (provided with USER enzyme) at 1:1 ratio
- Add 1.4uL of USER:Cutsmart premix to each reaction well
- Incubate the mixture in a thermal cycler with following cycles:
- 15min@37°C
- Lid heated@50°C
- Step4) ProNext Bead purification/size selection
- Add 13.2uL of ProNext bead to 12uL of ligated DNA from step3 (1:1.1 ratio of ligated DNA to bead)
- Pipette mix 10 times and incubate@ room temp for 10min
- Place on magnetic stand for 2min
- Carefully remove and discard the supernatant
- Add 100uL washing buffer, wait for 30 seconds, discard washing buffer
- Repeat washing step one more time, remove any extra washing buffer, remove the plate/tube from magnetic stand and let air-dry for 5min
- Add 15uL of elution buffer
- Return to magnetic stand, wait for 1min, pipette the eluted DNA to new tubes/plate => keep at -20 or procced to next step
- Step5) Limited PCR to enhance library and attach barcodes

|  | x1 |
| --- | --- |
| Ligated DNA | 7.5uL |
| Q5 master mix | 12.5uL |
| i7 primer/barcode | 2.5uL |
| i5 primer/barcode | 2.5uL |

- Incubate the mixture in a thermal cycler with following cycles:

1. 30sec@98°C
2. 10sec@98°C
3. 75sec@65°C (repeat steps a & b for 7-8 cycles)
4. 5min@65°C

- Lid heated@105°C
- Step6) ProNext Bead purification and dual size selection
  - Add 18uL of ProNext bead to 20uL of PCR product from step3 (1:0.9 ratio of PCR to bead)
  - Pipette mix 10 times and incubate@ room temp for 8min
  - Place on magnetic stand for 2min
  - Transfer supernatant to new clean tubes (at this step very large unwanted DNA fragments are attached to beads and will be discarded)
  - Add 4uL of ProNext bead to new tubes containing the supernatant from previous step (1:0.2 ratio of original PCR product (20uL) to bead; this step removes any fragment below ~300bp)
  - Pipette mix 10 times and incubate@ room temp for 8min
  - Place on magnetic stand for 2min, discard the supernatant
  - Add 100uL washing buffer, wait for 30 seconds, discard washing buffer
  - Repeat washing step one more time, remove any extra washing buffer, remove the plate/tube from magnetic stand and let air-dry for 5min
  - Add 20uL of elution buffer
  - Return to magnetic stand, wait for 1min, pipette the eluted DNA to new tubes/plate => keep at -20 or procced to QC and sequencing
- Libraries at this step, after dual size selection, are all in the same size distribution (~800bp) and can therefore be normalized based on DNA concentration measured by a fluorometer assay (e.g. Qubit or PicoGreen assay). However, we suggest that prior to combining libraries together, check the quality/size of few random libraries on BioAnalyzer to make sure of the performance of the protocol.
